# Role of Extracellular Vesicles in Amyotrophic Lateral Sclerosis

**DOI:** 10.3389/fnins.2018.00574

**Published:** 2018-08-17

**Authors:** Deborah Ferrara, Laura Pasetto, Valentina Bonetto, Manuela Basso

**Affiliations:** ^1^Laboratory of Transcriptional Neurobiology, Centre for Integrative Biology (CIBIO), University of Trento, Trento, Italy; ^2^Department of Molecular Biochemistry and Pharmacology, Istituto di Ricerche Farmacologiche Mario Negri IRCCS, Milan, Italy

**Keywords:** extracellular vesicles, amyotrophic lateral sclerosis, biomarkers, prion-like behavior, therapy

## Abstract

Amyotrophic Lateral Sclerosis (ALS) is the most common motor neuron disease in adults and primarily targets upper and lower motor neurons. The progression of the disease is mostly mediated by altered intercellular communication in the spinal cord between neurons and glial cells. One of the possible ways by which intercellular communication occurs is through extracellular vesicles (EVs) that are responsible for the horizontal transfer of proteins and RNAs to recipient cells. EVs are nanoparticles released by the plasma membrane and this review will describe all evidence connecting ALS, intercellular miscommunication and EVs. We mainly focus on mutant proteins causing ALS and their accumulation in EVs, along with the propensity of mutant proteins to misfold and propagate through EVs in prion-like behavior. EVs are a promising source of biomarkers and the state of the art in ALS will be discussed along with the gaps and challenges still present in this blooming field of investigation.

## Introduction

Amyotrophic Lateral Sclerosis (ALS) is the most common motor neuron disease, with a prevalence of five cases per 100,000 persons. It is a rare and fatal neurodegenerative disorder that primarily targets upper and lower motor neurons in the motor cortex, brainstem, and spinal cord. It results in progressive paralysis and death, generally within three to 5 years from onset, although the progression is slower in some patients (Hardiman et al., 2017). Genetic mutations associated with ALS have been observed both in sporadic cases, where the mutation may occur *ex novo*, and in familial cases, where the mutation is inherited from generation to generation. Most ALS cases are sporadic, and 10% are familial, almost always as a dominant trait (**Table 1**).

**Table 1:** List of genes implicated in ALS pathogenesis, according to OMIM.

Gene/Locus	Location	Phenotype	Inheritance	ExoCarta	References^∗^
*TARDBP*	1p36.22	ALS/FTD	AD	Y	Ding et al., 2015; Feiler et al., 2015; Iguchi et al., 2016
*ALS2*	2q33.1	ALS, juvenile	AR	N	
*ERBB4*	2q34	ALS	AD	Y	
*TUBA4A*	2q35	ALS/FTD	AD	Y	
*CHMP2B*	3p11.2	ALS	AD	Y	
*MATR3*	5q31.2	ALS	AD	Y	
*SQSTM1*	5q35.3	ALS/FTD	AD	Y	
*FIG4*	6q21	ALS	AD	N	
*C9orf72*	9p21.2	ALS/FTD	AD	N (DPR)	Westergard et al., 2016
*SIGMAR1*	9p13.3	ALS, juvenile	AR	N	
*VCP*	9p13.3	ALS/FTD		Y	
*SETX*	9q34.13	ALS, juvenile	AD	N	
*OPTN*	10p13	ALS		Y	
*ANXA11*	10q22.3	ALS	AD	Y	
*HNRNPA1*	12q13.13	ALS	AD	Y	
*TBK1*	12q14.2	ALS/FTD	AD	Y	
*ANG*	14q11.2	ALS		Y	
*SPG11*	15q21.1	ALS, juvenile	AR	Y	
*FUS*	16p11.2	ALS/FTD		Y	Kamelgarn et al., 2016
*PFN1*	17p13.2	ALS		Y	
*ALS3*	18q21	ALS	AD	N	
*ALS7*	20p13	ALS		N	
*VAPB*	20q13.32	ALS	AD	Y	
*SOD1*	21q22.11	ALS	AD, AR	Y	Gomes et al., 2007; Basso et al., 2013; Grad et al., 2014
*CHCHD10*	22q11.23	ALS/FTD	AD	N	
*UBQLN2*	Xp11.21	ALS/FTD	XLD	Y	

*Y is the abbreviation for YES, N for NO. ^∗^References are related to EV protein cargos described in the paragraph “ALS-associated proteins as EV cargo and the prion-like propagation of protein misfolding.” AD, autosomal dominant; AR, autosomal recessive; XLD, X-linked disease*.

In 1993 the first genetic mutation found to cause ALS was reported in the gene SOD1, which encodes the superoxide-dismutase protein (Rosen et al., 1993). More than thirty genes have been linked to ALS, but causality has been validated only for about fourteen (Taylor et al., 2016) (**Table 1**). These genes are involved in proteostasis and protein quality control (e.g., *SOD1, VCP, OPTN, UBQLN2, SQSTM1, TBK1*) (Rosen et al., 1993; Johnson et al., 2010; Maruyama et al., 2010; Deng et al., 2011; Fecto et al., 2011; Freischmidt et al., 2015), RNA processing and metabolism (e.g., *AGN, TARDBP, FUS, C9ORF72, HNRNPA1, MATR3*) (Greenway et al., 2006; Sreedharan et al., 2008; Kwiatkowski et al., 2009; DeJesus-Hernandez et al., 2011; Renton et al., 2011; Kim et al., 2013; Johnson et al., 2014) and cytoskeletal dynamics (e.g., *DCTN1, TUBA4A*) (Munch et al., 2004; Smith et al., 2014) which are impaired in ALS. Interestingly, many of the mutant genes cause either ALS or another neurodegenerative disease named frontotemporal dementia (FTD), or a degenerative condition with common traits of ALS and FTD (Nguyen et al., 2018). A typical hallmark of the pathology are the protein inclusions that are often ubiquitinated and enriched in TDP-43 (Neumann et al., 2006). Another characteristic of ALS is illustrated by clinical evidence that the disease begins focally and spreads during progression, propagating motor neuron death from one starting motor unit to adjacent ones (Brettschneider et al., 2015), possibly through a mechanism involving altered intercellular communication between neurons and glial cells (Garden and La Spada, 2012).

## ALS Is a Non-Cell Autonomous Disease

Amyotrophic lateral sclerosis is a non-cell autonomous disease, and motor neuron degeneration is modulated by intracellular and intercellular damages (Ilieva et al., 2009). Elegant studies have demonstrated that the knock-out or reduction, respectively, of mutant SOD1 or TDP-43 selectively in motor neurons delayed disease onset but did not affect its progression (Clement et al., 2003; Ditsworth et al., 2017). In contrast, the selective removal of mutant SOD1 from microglia or astrocytes *in vivo* slowed the progression of the disease (Beers et al., 2006; Boillee et al., 2006; Yamanaka et al., 2008; Wang et al., 2009), giving glial cells a central role in disease propagation (Ilieva et al., 2009).

Lymphocytes may serve as another layer of regulation because removal of CD4+ T lymphocytes in an ALS mouse model increased disease severity by impairing the trophic responses of microglia and astrocytes (Beers et al., 2008). Moreover, reduction of regulatory T-lymphocyte numbers and function correlates with faster progression and greater severity in patients (Beers et al., 2011; Beers et al., 2017).

Finally, overexpression of mutant SOD1 in skeletal muscle affected motor neuron viability and suggested that the physical communication between skeletal muscle and nerve influenced neuronal survival, axonal growth, and maintenance of synaptic connections in ALS (Dobrowolny et al., 2008). These data suggest that interactions between different cell population are affected.

## Intercellular Communications, Unconventional Protein Secretion and Extracellular Vesicles

In the Central Nervous System (CNS), intercellular crosstalk happens among neurons, between neurons and glia or cells of the innate immune system, through different modalities, involving the release into the extracellular space of molecules such as neurotransmitters, neurotrophic factors, metabolites, and mutant proteins encapsulated or not in vesicles (Fevrier et al., 2004; Verkhratsky and Toescu, 2006; Basso and Bonetto, 2016; Garden and La Spada, 2012; Verkhratsky et al., 2016).

Proteins are secreted by either a classical or an unconventional modality. The classical, or conventional, protein secretion refers to the transport of secreted proteins through the endoplasmic reticulum (ER), to the endosomes, the Golgi and then to the plasma membrane, if appropriately folded. Eventually, immature cargos are efficiently transported back to the ER. The transport of proteins between organelles within the secretory pathway occurs via vesicles characterized by different sets of cytosolic proteins that generate distinct classes of transport vesicles. There are three principal categories characterized by evolutionarily related coat proteins, named clathrin, COPI, and COPII. The clathrin-coated vesicles are necessary to transport vesicles between the plasma membrane and the *trans*-Golgi Network to fuse with endosomes or lysosomes; COPI-coated vesicles transport cargo from the Golgi back to the ER. COPII vesicles transport cargo proteins from the ER to the Golgi [for a detailed review on the conventional protein secretion, refer to Gomez-Navarro and Miller (2016)]. With unconventional protein secretion (UPS), proteins that do not present a signal peptide for secretion (leaderless proteins) are released into the extracellular space. UPS occurs by different modes: it is usually induced by cell stress, and also involves the transport of proteins to the plasma membrane in vesicular intermediates such as CUPS (compartment for unconventional protein secretion), late endosomes, secretory autophagosomes, and lysosomes (Malhotra, 2013; Dimou and Nickel, 2018). Once the leaderless proteins reach the plasma membrane, they are released into the extracellular space as free cargos. In addition to the UPS pathways, leaderless proteins reach the extracellular space through extracellular vesicles (EVs) (Rabouille, 2017). These include exosomes, microvesicles, and apoptotic bodies, differing in size, biogenesis and mechanism of secretion (Raposo and Stoorvogel, 2013; Baixauli et al., 2014). In the CNS, all cell types release EVs which contain proteins, RNA and metabolites (Tkach and Thery, 2016). Exosomes are small vesicles (50–100 nm) and derive from the inward budding of endosomal multivesicular bodies (MVBs). In the early endosomes, ubiquitinated proteins are recognized by the endosomal sorting complexes required for transport (ESCRT) machinery and targeted to the late endosomes, in the intraluminal vesicles (ILV) of the MVBs (Colombo et al., 2013).

Endosomal sorting complexes required for transport-independent mechanisms have now been reported for exosome biogenesis in ILV and they are based on the interaction of the cargo proteins with synthenin, syndecan, and ALG-2 interacting protein X (ALIX) (Baietti et al., 2012). By mechanisms still under investigation, MVBs either fuse with the lysosome for protein degradation or transit to the plasma membrane for exosome release. Recent data indicated that specific protein post-transcriptional modifications, called ISGylation, inhibits EV release and induces lysosome degradation (Villarroya-Beltri et al., 2016). Accordingly, lysosome inhibition correlates with increased release of alpha-synuclein from SH-SY5Y cells (Alvarez-Erviti et al., 2011), supporting the hypothesis that if lysosome is impaired, EVs can be used as a vehicle for the disposal of unwanted material. Defects in the endolysosomal pathway have been observed in Alzheimer’s disease (AD) models, resulting in aberrant release of Amyloid Precursor Protein (APP) species in exosomes, allowing for elimination of lysosomal contents that cannot be efficiently degraded (Miranda et al., 2018). C9orf72, which presents aberrant hexanucleotide (GGGGCC) expansion in the non-coding region in ALS patients, regulates vesicle trafficking (Aoki et al., 2017; Farg et al., 2017). The expansion reduces the interaction between C9orf72 and Rab7L1. Rab7L1 is a regulator of vesicle transport from the MVB to the plasma membrane. The reduced interaction between expanded C9orf72 and Ran7L1 correlates with a diminished amount of released EVs (Farg et al., 2017). The mechanisms responsible for impaired EV release are not yet clear but recent reports indicate that expansion of C9orf72 also alters lysosome degradation. Other genes linked to ALS, namely *FIG4, ALS2, CHMP2B, OPTN, SQSTM1*, are involved in endosomal maturation, lysosome biogenesis and vesicle trafficking (Shi et al., 2018), suggesting this pathway as pathogenic in ALS.

## ALS-Associated Proteins as EV Cargo and the Prion-Like Propagation of Protein Misfolding

**FIGURE 1:**
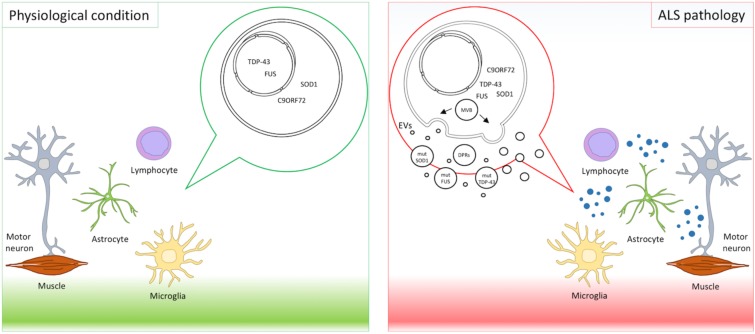
EVs in ALS pathology. Motor neurons, astrocytes, microglia, lymphocytes, and muscles interplay and contribute to ALS pathogenesis. Mutant proteins implicated in ALS, such as mutant SOD1, TDP-43, FUS, and DPRs derived from expanded C9orf72, were detected in EVs and suggest EVs as means of disease spreading.

Extracellular vesicles deliver cargos from donor to recipient cells. Pathogenic proteins such as prions, amyloid β peptide, superoxide dismutase, alpha-synuclein, and tau are released in association with EVs (Pimpinelli et al., 2005; Gomes et al., 2007; Emmanouilidou et al., 2010; Alvarez-Erviti et al., 2011; Saman et al., 2012; Yuyama et al., 2015) displaying a prion-like behavior and propagating the disease (Ghidoni et al., 2008; Coleman and Hill, 2015). Mutant proteins causative in ALS have been retrieved in EVs and are transferred across the brain cells as a means of spreading the disease (Silverman et al., 2016) (**Figure 1**).

SOD1, or superoxide dismutase one, is an abundant enzyme that converts superoxide molecules into hydrogen peroxide and dioxygen. SOD1 was the first ALS-associated protein to be detected in EVs from stable mouse motor neuron-like (NSC-34) cells that overexpressed human wild-type and mutant SOD1 (Gomes et al., 2007). Subsequently, we proved that ALS astrocytes release mutant SOD1 both trough protein secretion and EVs. We exposed wild-type motor neurons to either the astrocyte secretome depleted of EVs or to purified EVs. Only purified EVs transmitted mutant SOD1 and induced the death of wild-type motor neurons, suggesting that EVs are mediators of toxicity (Basso et al., 2013). SOD1 can self-replicate *in vitro* and transfer aggregates from cell to cell in culture (Sibilla and Bertolotti, 2017).

Grad and colleagues further characterized the aggregation state of mutant SOD1 in EVs and proposed that misfolded SOD1 in EVs contributes to the prion-like propagation of the pathology in the CNS (Grad et al., 2014). Misfolded SOD1 associated with the outer leaflet of exosomes in NSC-34 cells stably-expressing mutant or wild-type SOD1, and was propagated to naïve NSC-34 cells. Similarly, cells exposed to conditioned media derived from HEK293 cells overexpressing mutant SOD1 had intracellular accumulation of misfolded SOD1. However, the EV-depleted media failed to propagate misfolded SOD1, suggesting that it is mainly transmitted by EVs (Grad et al., 2014).

Another protein retrieved in EVs is TDP-43. This DNA and RNA binding protein mostly resides in the nucleus and has multiple functions in transcriptional repression, pre-mRNA splicing and translational regulation (Mackenzie et al., 2010). When mutated, it translocates to the cytosol and its cytoplasmic aggregation is a pathological hallmark of ALS and FTD (Scotter et al., 2015). TDP-43 presents a prion-like structure at the C-terminal domain of the protein sequence, that has a tendency to oligomerize and aggregate. With a protein complementation assay, Feiler and colleagues demonstrated that TDP-43 oligomers were loaded in EVs and taken up by the neuronal soma and the synaptic cleft, and contributed to neuronal degeneration (Feiler et al., 2015). Additionally, TDP-43 was enriched in the cerebrospinal fluid (CSF) of ALS-FTD patients (Ding et al., 2015). Treatment of U251 cells with ALS-FTD CSF for 21 days increased the accumulation of the toxic TDP-43 C-terminus fragments in cell lysate (Ding et al., 2015). The authors speculated that TDP-43 C-terminus fragments in CSF act as a “seed” to spread pathological TDP-43 in cultured cells, providing indirect evidence of TDP-43 prion-like behavior mediated by EVs. TDP-43 was also detected in EVs released by Neuro2a cells and primary neurons but not from astrocytes or microglia (Iguchi et al., 2016). The authors correlated TDP-43 loading in EVs as beneficial in neuronal clearance of pathological TDP-43 because inhibition of exosome secretion by inactivation of neutral sphingomyelinase two with GW4869 or by silencing RAB27A enhanced TDP-43 aggregates in Neuro2a cells and exacerbated the disease progression in a TDP-43 transgenic mouse model (Iguchi et al., 2016).

Fused in Sarcoma (FUS) is another nuclear RNA-binding protein implicated in a subset of familial and sporadic ALS cases. Like in TDP-43 pathology, FUS mutations induce cytoplasmic mislocalization and the formation of stress granule-like structures (Mackenzie et al., 2010). Kamelgarn and colleagues analyzed FUS interacting partners in Neuro2a and SHSY5Y cells and noted that 42 interacting partners were annotated in ExoCarta. By analyzing the EV cargos purified from cells overexpressing FUS-WT or mutant (R521G, R495X), FUS was detected in the EVs and particularly enriched in the FUS-R495X expressing cells (Kamelgarn et al., 2016), suggesting that FUS secretion might contribute to the cell-to-cell spreading of FUS pathology.

Finally, a recent paper described dipeptide repeat proteins (DPRs) derived from non-ATG translation (RAN-translation) of C9orf72 hexanucleotide repeat expansions in EVs (Westergard et al., 2016). Aberrant hexanucleotide repeat expansions in C9orf72 are the most common genetic alterations in ALS and FTD (Renton et al., 2014). DPRs were detected in the spinal cord of ALS patients (Gendron et al., 2013) and were shown to contribute to motor neuron degeneration (Ash et al., 2013; Wen et al., 2014). In an elegant *in vitro* setting, using cell cultures in transwells and microfluidic fluid chambers, NSC34 transfected with DPRs and spinal motor neurons derived from induced pluripotent stem cells from C9orf72-ALS patients released DPRs in EVs. Intercellular transmission of DRPs happened both through anterograde and retrograde transport in neurons, similarly to TDP-43 (Feiler et al., 2015), and also between neurons and astrocytes. These data provided additional evidence that EVs contribute to disease spreading. Unconventional DPR secretion was also reported but the causative role in neuronal death was not explored (Westergard et al., 2016). These results underline the importance of EVs in spreading toxic proteins in the CNS cellular environment and in contributing to the propagation of ALS (**Figure 1**).

## ALS-Associated RNA as EV Cargo

Extracellular vesicles are responsible for the horizontal transfer not only of proteins but also of RNAs to recipient cells (Fruhbeis et al., 2013), and the EV-transferred RNA (exRNA) is functionally active (Lai et al., 2015; Tkach and Thery, 2016). RNA enriched in EVs comprises mostly small non-coding RNA like microRNA, rRNA, tRNA (also called tRFs, tRNA-derived RNA fragments), YRNA, snRNA, snoRNA, lncRNA and vault RNA (Mateescu et al., 2017). This intercellular transmission of RNA is of interest for ALS pathology because of recent evidence pointing to a fundamental role for RNA and RNA-binding protein dyshomeostasis as crucial in this disease (Donnelly et al., 2014). In fact, several pathogenic mutations in ALS occur in genes involved in RNA processing and activity (*ANG, TARDBP, FUS, ATXN2, TAF15, MATR3*) (Peters et al., 2015) and selective knockout of Dicer, the critical enzyme in microRNA maturation, leads to motor neuron death (Haramati et al., 2010). Accordingly, miRNA 218 is one of the most abundant RNA in motor neurons, and its knockout leads to neuromuscular failure and motor degeneration (Amin et al., 2015).

Several studies have investigated miRNA in CSF, urine, serum or plasma of ALS patients but no biomarkers are available yet for this disease, probably because of the technical variability in the analysis of circulating miRNA (Grasso et al., 2015). EVs offer an attractive alternative because small RNAs are protected by the EV lipid bilayer; several laboratories are working to define biomarkers from EVs, but data are mostly not yet available for ALS [for a broader review of RNAs in EVs, refer to Basso and Bonetto (2016)].

Pinto et al. (2017) analyzed EVs purified from NSC-34 cells overexpressing SOD1-WT and G93A for the RNA content. EVs derived from NSC-34 overexpressing SOD1-G93A transmitted increased levels of miR-124 to microglia N9 cells and halved their phagocytic ability. EVs also led to persistent NF-kB activation as well as upregulation of genes involved in the activation of microglia (Pinto et al., 2017). Conversely, in EVs isolated from astrocyte primary cultures derived from SOD1-WT and G93A no difference was seen between controls and mutant EVs in miRNA cargos (Jovicic and Gitler, 2017), while the toxicity of mutant EVs on motor neurons was confirmed, as previously reported (Basso et al., 2013). These different results may be linked to methodological challenges in EV purification and library preparations for RNA-seq. Positional papers from the International Society of Extracellular Vesicles (ISEV) are available to standardize the techniques across laboratories to improve reproducibility (Mateescu et al., 2017).

## Extracellular Vesicles as Biomarkers in ALS

No biomarkers are available yet in ALS. This lack limits the classification of the stage of illness, delaying development of specific therapeutic interventions. EVs are potentially an attractive source of biomarkers. They are released continuously into biofluids (CSF and blood) and carry biomolecular signatures (protein and nucleic acids) that reflect the pathological state of the cells from which they derive. Despite remarkable advances in the field, there are still technical issues that have to be overcome before EVs arrive in the clinic. First of all, EV isolation needs to become straightforward, rapid and cost-effective, with pre-analytical procedures that preserve EV structural and molecular integrity. Second, there is pressing need for gold standards for instruments and assays for detection of EV-associated biomarkers, as discussed in the latest ISEV workshop on the topic “EVs as disease biomarkers” (Clayton et al., 2018). In ALS, few data are publicly available on biological fluids from patients. In a case report, a dramatic increase of leukocyte-derived EVs in the CSF of one ALS patient was reported compared to healthy controls (Zachau et al., 2012). Subsequently TDP-43 was detected in exosomes derived from CSF in a limited cohort of ALS and FTD patients, and its expression was not significantly different from controls, although it tended to be higher in FTD patients (Feneberg et al., 2014). Later, TDP-43 full-length and C-terminus fragments were reported in a cohort of ALS-FTD patient-derived EVs of CSF origin (Ding et al., 2015).

Blood is the most attractive source of biomarkers because it involves only a minimally invasive medical procedure for patients and is potentially very informative. In fact, the discovery of exosomal-like vesicles in human blood plasma opened up new opportunities for biomarker discovery (Caby et al., 2005). Blood EVs derive mainly from erythrocytes and platelets, but a lower percentage also comes from endothelial cells and leukocytes (Shet et al., 2003; Nielsen et al., 2014). As mentioned earlier, ALS is a non-cell autonomous and multisystem disease. Therefore, blood EVs could deliver information on early pathological events. Tomlinson et al., in a proteomic analysis of serum EVs from Parkinson’s disease and ALS patients, found 54 proteins that could discriminate between the two groups of patients (Tomlinson et al., 2015). Several of the proteins identified in this and other proteomic studies are abundant, deriving from blood, and associate with EVs, as reported in EVpedia, ExoCarta, Vesiclepedia, and Plasma Proteome Database, the major curated databases on EVs (Mathivanan and Simpson, 2009; Kalra et al., 2012; Nanjappa et al., 2014; Kim et al., 2015). The high contamination from blood proteins, however, hinders a more profound identification of the EV proteome, and shows that analyses of EV cargos isolated from biofluids are still challenging. Possibly, to overcome aspecificity, EV subpopulations should be immunopurified. In a recently published approach, a two-step procedure to select for neuronal or astrocytic surface markers allowed the isolation of EVs enriched for neuronal or astrocytic origin, respectively (Mustapic et al., 2017). Preliminary data in Alzheimer’s and FTD patients demonstrated good diagnostic and predictive performance for this method (Goetzl et al., 2015, 2016a,b, 2018).

In conclusion, EV-associated cargos hold promise as biomarkers for neurodegenerative diseases, however, a deep information gap still need to be filled in ALS, where no study on EV RNA cargos has been published yet. Proteomics combined with other omics screenings such as lipidomics, metabolomics, and transcriptomics will probably soon identify useful candidate biomarkers to be validated in extensive clinical studies, while standard operating procedures for collection, storage, handling and analysis of EVs are still urgently awaited.

## Conclusion: Extracellular Vesicles as Therapeutic Opportunities in ALS

Extracellular vesicles are microparticles that hold out potentials for pathogenesis investigation and biomarker discovery. Important work has been recently published on the propensity of EVs to propagate misfolded proteins from cell to cell in ALS but thorough information on EV cargos, such as metabolites, lipids, and RNA, is still lacking. A more recent application of EVs is therapeutics. EVs derived from murine adipose-derived stromal cells protected NSC-34 cells expressing ALS mutations against oxidative stress-dependent damage (Bonafede et al., 2016). Murine adipose-derived stromal cells EVs reduced cytosolic SOD1 and ameliorated mitochondrial abnormalities (Lee et al., 2016), and were proposed to attenuate the disease. These promising preliminary data hold hope for the future but highlight the need for more and deeper investigations in the field.

## Author Contributions

VB and MB discussed and organized the paper. DF, LP, VB, and MB wrote the paper. DF prepared the figure.

## Conflict of Interest Statement

The authors declare that the research was conducted in the absence of any commercial or financial relationships that could be construed as a potential conflict of interest.
